# Molecular epidemiological survey of bacteremia by multidrug resistant *Pseudomonas aeruginosa*: the relevance of intrinsic resistance mechanisms

**DOI:** 10.1371/journal.pone.0176774

**Published:** 2017-05-08

**Authors:** Raquel Cristina Cavalcanti Dantas, Rebecca Tavares e Silva, Melina Lorraine Ferreira, Iara Rossi Gonçalves, Bruna Fuga Araújo, Paola Amaral de Campos, Sabrina Royer, Deivid William da Fonseca Batistão, Paulo Pinto Gontijo-Filho, Rosineide Marques Ribas

**Affiliations:** 1 Institute of Biomedical Sciences, Laboratory of Molecular Microbiology, Federal University of Uberlandia, Uberlandia, Minas Gerais, Brazil; 2 Institute of Biomedical Sciences, Laboratory of Trypanosomatids/Molecular Biology, Federal University of Uberlandia, Uberlandia, Minas Gerais, Brazil; 3 School of Medicine, Federal University of Uberlandia, Uberlandia, Minas Gerais, Brazil; University of North Dakota, UNITED STATES

## Abstract

The bacterial factors associated with bacteremia by multidrug-resistant and extensively drug-resistant *P*. *aeruginosa*, including overexpression of efflux pumps, AmpC overproduction, and loss/alteration of the OprD porin in isolates that are non-Metallo-β-Lactamase producing were analyzed in a retrospective study. Molecular analyses included strain typing by Pulsed Field Gel Electrophoresis and identification of key genes via qualitative and quantitative PCR-based assays. Previous use of carbapenems and tracheostomy was independently associated with the development of bacteremia by extensively drug-resistant and multidrug-resistant strains of *P*. *aeruginosa*. A high consumption of antimicrobials was observed, and 75.0% of the isolates contained amplicons with the *bla*_SPM-1_ and *bla*_VIM_ genes. Of the 47 non-Metallo-β-Lactamase isolates, none had another type of carbapenemase. However, the isolates exhibited high rates of hyperproduction of AmpC, loss of the OprD porin (71.4%) and the presence of MexABOprM (57.1%) and MexXY (64.3%). This study suggests that in non-Metallo-β-Lactamase isolates, the association of intrinsic resistance mechanisms could contributes to the expression of multidrug-resistant/extensively drug-resistant phenotypes.

## Introduction

*Pseudomonas aeruginosa*, an opportunistic pathogen, is an major cause of health care associated infections [1,2], mainly in patients with impaired immune systems [1]. Today, high levels of resistance to several antimicrobial agents in *P*. *aeruginosa* clinical isolates have been reported as a worldwide problem [3–5]; however, this problem is more significant in Brazil due to a very high frequency of antibiotic use, particularly carbapenems and fluoroquinolones [6,7].

The carbapenems remain the primary antimicrobial used for treatment of severe infections caused by *P*. *aeruginosa*, however, the emergence and spread of resistance to these antibiotics may compromise their efficacy and are thus associated with high rates of mortality [8–11].

One of the most common resistance mechanisms to carbapenems in *P*. *aeruginosa* isolates is the loss or alteration of the outer membrane porin protein OprD [12], which regulates the entry of this class of antibiotics into the cell [13]. Other mechanisms include the upregulation of efflux pump systems, such as MexAB-OprM, MexEF-OprN, MexCD-OprJ and MexXY-OprM, the production of carbapenemase enzymes, including Metallo-β-lactamases (MBLs), especially the SPM-1 variant in Brazil, OXA-type carbapenemases and class A carbapenemases (e.g., GES and KPC types), and the overexpression of extended-spectrum chromosomally encoded AmpC cephalosporinase [12,14].

Many studies examining the mechanisms of carbapenem-resistance have involved controlled laboratory-derived strains; however, studies involving clinical isolates have not clearly defined the contributions of the MBL, AmpC cephalosporinase, alterations of the OprD porin or efflux systems to carbapenem-resistance [11,15]. In this report, we analyzed the mechanisms of carbapenem-resistance in clinical isolates of multidrug-resistant *P*. *aeruginosa* from blood samples. In addition, we evaluated the dissemination of high-risk clones in hospital settings.

## Materials and methods

### Patients and setting

The clinical microbiology laboratory database was reviewed, and 157 patients with nosocomial *P*. *aeruginosa* bacteremia from May 2009 to December 2012 at Uberlandia University Hospital (Brazil) were identified. Only the first episode of bacteremia was analyzed for those patients with more than one episode.

### Study design and data collection

A retrospective observational cohort study was employed to identify the risk factors of multidrug-resistant (MDR) or extensively drug-resistant (XDR) *Pseudomonas aeruginosa* bacteremia. The demographic, clinical and epidemiological characteristics of the patients were obtained from individual medical records, following the model of the NHSN (National Healthcare Safety Network). For each patient studied, the following characteristics were recovered from their clinical records: age, gender, hospitalization time ≥ 30 days prior to infection, admission to the ICU, surgery, previous antibiotic use, invasive procedures such as mechanical ventilation, tracheostomy, urinary catheter, central venous catheter, surgical drain during the current hospitalization, enteral catheter or gastric nutrition, hemodialysis and parenteral nutrition.

### Definitions and DDD for antibiotics

According to the Centers for Disease Control and Prevention [16], bacteremia can be defined as the presence of viable bacteria in the blood documented by a positive blood culture result. Bacteremia is considered as nosocomial if the infection occurred more than 48 hours after admission and if there was no clinical evidence of infection at the time of admission [17]. The MDR and XDR strains were defined according to Magiorakos and collaborators [18]. Previous antibiotic use was considered when the patient received therapy with any antibiotic for at least 72 hours during a period of 30 days prior to the microbiological infection diagnosis [19]. Antimicrobial consumption was expressed in a Defined Daily Dose (DDD) [20] per 1000 patient-days, and the data collection was conducted in the monthly electronic reports of antibiotic dispensing provided by the hospital pharmacy. The antimicrobial agents that were tracked included the following: cephalosporins (ceftriaxone and cefepime), carbapenems (imipenem and meropenem) and fluoroquinolones (ciprofloxacin and levofloxacin).

### Bacterial isolates and clinical microbiology

A total of 157 consecutive non-duplicate *P*. *aeruginosa* isolates from patient blood were included in the study. Cultures were processed using a BACT/Alert (bioMérieux). Microbial identification and antimicrobial susceptibility test were performed on a VITEK II automated system (bioMérieux) for the following antimicrobials: aminoglycosides (gentamicin, amikacin), carbapenems (imipenem, meropenem), cephalosporins (ceftazidime, cefepime), fluoroquinolones (ciprofloxacin, levofloxacin), penicillins plus beta-lactamase inhibitors (piperacillin-tazobactam), monobactams (aztreonam) and polymyxins (colistin, polymyxin B). The isolates with intermediate susceptibility were considered as resistant. Quality control protocols were used according to the standards of the Clinical and Laboratory Standard Institute [21–23]. Fifty-six carbapenem-resistant *P*. *aeruginosa* isolates, confirmed by VITEK II system, were phenotypically screened for MBL production using a Double-Disc Synergy Test (DDST), as previously described [24]. The isolates that showed intermediate or resistant zones of inhibition for imipenem were tested for carbapenemase production with a Modified Hodge Test (MHT) according to CLSI recommendations and quality control [22–23]. The AmpC β-lactamase production was performed with an AmpC Disk Test that is based on the use of Tris-EDTA to permeabilize a bacterial cell and release β-lactamases into the external environment [25].

### Molecular microbiology testing

The DNA extraction was performed by the technique of thermal lysis. A conventional multiplex PCR assay was performed for 56 isolates to detect five families of the MBL genes (*bla*_IMP_, *bla*_VIM_, *bla*_SPM_, *bla*_GIM_, *bla*_SIM_), using previous published primers [26]. The cycling conditions of this PCR were as follows: 94°C for 5 min, followed by 30 denaturation cycles at 94°C for 30 sec; annealing at 53°C for 45 sec; and extension at 72°C for 30 sec, followed by final extension at 72°C at 10 min. The strains were then submitted to a new conventional multiplex PCR that targets class D carbapenemases such as oxacillinase (*bla*_OXA-51_, *bla*_OXA-23_, *bla*_OXA-40_ and *bla*_OXA-58_) [27]. Additionally, the *bla*_KPC_ gene was detected by conventional PCR using primers and cycling conditions previously described [27]. The amplified PCR products were visualized by electrophoresis on 1.5% agarose gels using the photo documentation System L-Pix EX (Loccus Biotechnology, Brazil).

### Pulsed-field gel electrophoresis (PFGE)

Nine MBL-positive *P*. *aeruginosa* isolates (9/56) were typed by pulsed-field gel electrophoresis (PFGE) according to the protocols described by Galetti [28] with modifications, following digestion of intact genomic DNA with the restriction enzyme *Spe*I (Promega, Brazil). DNA fragments were separated on 1% (w/v) agarose gels in TBE 0.5% [Tris–borate–ethylene diamine tetra-acetic acid (EDTA)] buffer using a CHEF DRIII apparatus (Bio-Rad, USA) with 6 V/cm, pulsed from 5 s to 40 s, for 21 hours at 12°C. Gels were stained with ethidium bromide and photographed under ultraviolet light. Computer-assisted analysis was performed using BioNumerics 5.01 software (Applied Maths, Belgium). We were unable to determine the macrorestriction analysis profile of a single *P*. *aeruginosa* strain containing the *bla*_SPM_ gene due to degradation of DNA. Comparison of the banding patterns was accomplished by the unweighted pair-group method with arithmetic averages (UPGMA) using the Dice similarity coefficient.

### Relative quantification of mRNA by real time PCR (RT-PCR)

Semi-quantitative RT-PCR was performed for 14 MBL-negative isolates with VeriQuest Fast SYBR Green qPCR Master Mix (Affymetrix®) using specific primers for the chromosomal genes *mexB*, *mexD*, *mexF*, *mexY*, *ampC* and *oprD*, as previously described with some modifications [26]. The reaction was prepared in a final volume of 25 μL, containing 10 pmol of each primer and 2 μL of cDNA. Amplification was carried out in triplicate from cDNA preparations (Applied Biosystems, model 7300®). The relative transcript levels were calculated according to the ΔΔCt method, as previously described [29,30]. Briefly, the ΔΔCt method provides the relative gene expression levels by averaging cycle threshold (Ct) values from triplicate RT-PCR reactions for target and housekeeping genes. The range of expression levels obtained for the triplicates, which incorporates the standard deviation (SD) of the ΔΔCt value into the fold-difference, was used to calculate the confidence interval (CI) considering a confidence level of 95%. Additionally, in order to minimize the error and preserve the accuracy and robustness of this test, we have excluded and repeated those triplicate reactions whose average of Ct values presented an SD value higher than 0.20. The *rps*L endogenous gene was used as the housekeeping gene and the wild-type *P*. *aeruginosa* PAO1 was used as the reference strain to determine the relative expression levels of the genes in the clinical isolates. The efflux systems MexAB-OprM, MexCD-OprJ, MexEF-OprN and MexXY-OprM were considered to be overexpressed when the transcriptional levels of *mexB*, *mexD*, *mexE* and *mexY* were at least two-, 100-, 100- and four-fold higher than those of the wild-type reference strain PAO1, respectively [26]. Reduced *oprD* expression and overexpression of *ampC* were considered relevant when their transcriptional levels were ≤ 70% and ≥ 10-fold, respectively, compared to the PAO1 reference strain [26].

### Statistical analysis

Student’s *t*-test was used to compare continuous variables and X^2^ or Fisher’s exact test was used to compare categorical variables. Variables with *P* ≤ 0.05 in the univariate analysis were candidates for multivariate analysis. All *P* values were two-tailed, and *P* values of 0.05 were considered statistically significant.

### Ethical considerations

The data and the samples analyzed in the present study were obtained in accordance with the standards and approved by the Federal University of Uberlandia Ethics Committee (UFU) through license number 00763112.7.0000.5152. For this study, samples were collected at the Microbiology Laboratory of the Clinical Hospital, with no contact with the patient and with the permission of the Hospital. This study was retrospective and there was no patient identification performed during data collection. Therefore, the ethics committee determined that informed consent was not required.

## Results

A total of 157 non-repetitive patients with *P*. *aeruginosa* bacteremia at the university hospital were included in this study. The univariate analysis and independent risk factors associated with MDR and XDR *P*. *aeruginosa* bacteremia are summarized in **Table 1.** According to antimicrobial susceptibility testing results, MDR and XDR *P*. *aeruginosa* bacteremia occurred in 67 (42.7%) and 35 (22.3%) of the cases, respectively. Clinical and demographic data from these patients (MDR and XDR) were compared with a sensitive *P*. *aeruginosa* infections group (non-MDR) (Table 1). In the whole series, prior exposures to carbapenems and having a tracheostomy were associated with the development of bacteremia by MDR or XDR *P*. *aeruginosa*. Intensive Care Unit admission and length of stay ≥ 30 days prior to infection were other independent risk factors to MDR *P*. *aeruginosa* bacteremia.

**Table 1:** Univariate analysis and independent risk factors associated with multidrug-resistant and extensively drug-resistant *P*. *aeruginosa* bacteremia.

Risk factor	XDR^1^ N = 35 (%)	MDR^2^ N = 67 (%)	Sensitive N = 90 (%)	XDR		MDR	
				Univariate *P*^4^ (OR^5^)	Multivariate *P* (OR)	Univariate *P* (OR)	Multivariate *P* (OR)
**Age–mean, years (range)**	56.06 (2–89)	56.58 (2–89)	49.09 (0–88)	0.42 (-)	-	0.10 (-)	-
**Gender Male**	22 (62.9)	44 (65.7)	61 (67.8)	0.75 (0.80)	-	0.91 (0.91)	-
**Gender Female**	13 (37.1)	23 (34.3)	29 (32.2)	0.75 (1.24)	-	0.91 (1.10)	-
**Hospitalization time ≥ 30 days prior to infection**	17 (48.6)	31 (46.6)	32 (35.6)	0.25 (1.71)	-	0.02 (2.19)*	0.05 (1.95)*
**ICU**^**3**^ **admission**	17 (48.6)	39 (58.2)	35 (38.9)	0.43 (1.48)	-	0.02 (2.19)*	0.02 (2.19)*
**Surgery**	19 (54.3)	34 (50.7)	35 (38.9)	0.17 (1.87)	-	0.18 (1.62)	-
**Previous antibiotic use**	31 (88.6)	57 (85.1)	67 (74.4)	0.13 (2.66)	-	0.01 (2.81)*	0.57 (1.29)
**Cephalosporin(3**^**rd**^ **generation)**	13 (37.1)	29 (43.3)	39 (43.3)	0.66 (0.77)	-	0.87 (1.00)	-
**Cefepime**	12 (34.3)	26 (38.8)	35 (38.9)	0.78 (0.82)	-	0.84 (1.00)	-
**Carbapenems**	22 (62.9)	39 (58.2)	37 (41.1)	0.04 (2.42)*	0.001 (3.48)*	0.05 (2.00)*	0.02 (2.22)*
**Fluoroquinolones**	7 (20.0	13 (19.4)	7 (7.8)	0.05 (2.96)*	0.51 (0.71)	0.05 (2.85)*	0.43 (1.42)
**Aminoglycosides**	4 (11.4)	5 (7.5)	7 (7.8)	0.49 (1.53)	-	0.81 (0.96)	-
**Invasive**	33 (94.3)	62 (92.5)	77 (85.5)	0.23 (2.79)	-	0.23 (2.09)	-
**Mechanical ventilation**	24 (68.6)	45 (67.2)	44 (48.9)	0.04 (2.28)*	0.61 (0.77)	0.03 (2.14)*	0.39 (1.42)
**Tracheostomy**	22 (62.9)	39 (58.2)	31 (34.4)	0.007 (3.22)*	0.02 (2.97)*	0.005 (2.55)*	0.05 (2.19)*
**Urinary catheter**	27 (77.1)	49 (73.1)	52 (57.8)	0.07 (2.52)	-	0.06 (1.99)	-
**Catheter central line**	30 (85.7)	54 (80.6)	71 (78.9)	0.53 (,61)	-	0.95 (1.11)	-
**Drain**	8 (22.8)	10 (15.0)	14 (15.6)	0.07 (2.36)	-	0.90 (0.95)	-
**Gastric/enteral tube**	24 (68.6)	50 (74.6)	59 (65.6)	0.91 (1.15)	-	0.29 (1.55)	-
**Haemodialysis**	13 (37.1)	21 (31.3)	18 (20.0)	0.07 (2.36)	-	0.14 (1.83)	-
**Parenteral nutrition**	4 (11.4)	9 (13.4)	16 (17.8)	0.55 (0.60)	—	0.60 (0.72)	-

^**1**^XDR, Extensively resistant

^2^MDR, Multidrug-resistant

^3^ICU, Intensive care unit

^**4**^*P *Value

^**5**^OR, Odds Ratio

*P ≤ 0.05 is statistically significant

**Fig 1:**
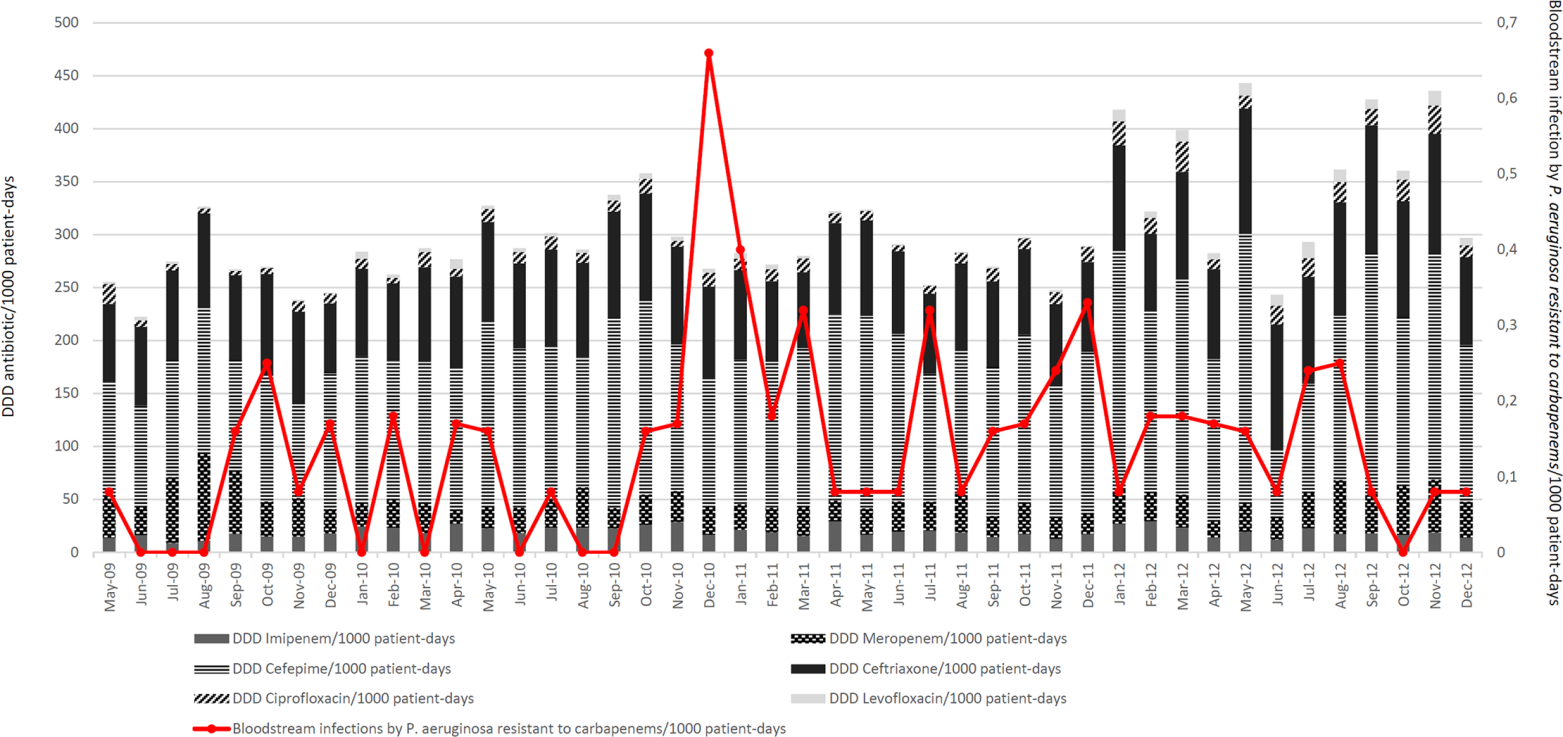
Endemic level of carbapenem resistant *P*. *aeruginosa* bacteremia per 1000 patient-days from March 2009 to December 2012.

The relationship between the defined daily dose of an antimicrobial per 1000 patient-days and the number of patients with carbapenem-resistant *P*. *aeruginosa* per 1000 patient-days are shown in **Fig 1.** Consumption of antimicrobials was high, and an increase in use was observed at the end of the study period, particularly for ceftriaxone, cefepime and meropenem. There was no positive correlation between the increase of carbapenem-resistant *P*. *aeruginosa* isolates and antimicrobial consumption.

All carbapenem-resistant *P*. *aeruginosa* isolates determined to contain MBL genes by PCR were subjected to molecular typing analysis. All these isolates were DDST positive. The genetic similarity among the strains was determined by PFGE and included the nine strains positive for the *bla*_SPM_ (six strains) and *bla*_VIM_ genes (three strains). It was impossible to determine the macro-restriction profile of one *bla*_SPM-_positive isolate due to degradation of the DNA sample. The genetic similarity dendrogram was generated from the macro-restriction profile, and the data from the *P*. *aeruginosa* with MBL genes are summarized in **Table 2.** In total, six different *P*. *aeruginosa* genotypic profiles were observed, differentiated from each other by a similarity factor above 80%. Two profiles corresponded to isolates containing the *bla*_SPM_ gene (profiles A and B) and another three profiles included the *bla*_VIM_ isolates (profiles C, D and E). The only A clone presented three subtypes (A1, A2 and A3) (**S1 Fig**, available in the Supporting Information). There was spread of clone A, as well as polyclonal dissemination of the *bla*_VIM_ isolates at the Hospital (**Table 2**). The antimicrobial susceptibility testing showed that all *P*. *aeruginosa* isolates analyzed by PFGE showed an MDR or XDR profile. The isolates from clone A exhibited the same resistance profile and were all classified as XDR, while pulsotype B was classified as MDR.

**Table 2:** Phenotypic and genotypic profile of resistance, hospital unit and the date of isolation of *P*. *aeruginosa* samples characterized by PFGE.

DDST^1^	*bla* Gene	Pulsotype PFGE^2^	Hospital Units	Date	MDR^3^/XDR^4^
**+**	**VIM**	E	Medical clinical	06/07/2011	XDR
**+**	**VIM**	D	Surgery unit	31/01/2011	XDR
**+**	**VIM**	C	Medical clinical	17/11/2010	MDR
**+**	**SPM**	B	Surgery unit	26/03/2011	MDR
**+**	**SPM**	A3	Surgery unit	07/08/2012	XDR
**+**	**SPM**	A2	Surgery unit	06/09/2011	XDR
**+**	**SPM**	A1	Adult ICU^5^	30/03/2012	XDR
**+**	**SPM**	A1	Adult ICU	10/09/2011	XDR

^**1**^Double Disk Synergistic Test

^2^PFGE; Pulsed-Field Gel Electrophoresis

^3^MDR, Multidrug-resistant

^**4**^XDR, Extensively-resistant

^**5**^ICU, Intensive Care Unit; + Positive test

Of the 56 carbapenem-resistant isolates, 47 did not show MBL production. Of this 47, 13 exhibited the MDR profile and 34 exhibited XDR profile and all 47 were included in the phenotypic and genotypic tests to search for other resistance mechanisms (**Table 3**). The Hodge test showed the presence of carbapenemase activity in just one isolate, which showed MDR/XDR profile, but the *bla*_OXA_ and *bla*_KPC_ genes were not detected by PCR in any isolate analyzed. The frequency of phenotypic production of the AmpC cephalosporin through the D-Test was common among isolates resistant to carbapenems (76.6%). However, the frequency of AmpC activity decreased in MDR strains (64.7%) and dropped further in XDR strains (38.4%).

**Table 3:** Characterization of resistance mechanisms in carbapenem-resistant *P*. *aeruginosa* isolates, both multidrug-resistant and extensively drug-resistant.

Phenotype	Total	Resistance Mechanisms					
		Carbapenemase		AmpC cephalosporinase		Loss of OprD	Efflux pump
		Hodge-test/ Positive (%)	PCR^1^/ Positive (%)	D-Test/ Positive (%)	qRT-PCR^2^*/ Positive (%)	qRT-PCR*/ Positive (%)	qRT-PCR*/ Positive (%) [system]
**Carbapenem resistant**	47	47/1 (2.1%)	47/0 (-)	47/36 (76.6)	14/10 (71.4)	14/10 (71.4)	14/8 (57.1) [ABM+] 14/9 (64.3) [XY+]
**MDR**^3^	34 (72.3)	34/1 (2.9)	34/0 (-)	34/22 (64.7)	10/6 (60.0)	10/6 (60.0)	10/7 (70.0) [ABM+] 10/9 (90.0) [XY+]
**XDR**^**4**^	13 (27.7)	13/1 (7.7)	13/0 (-)	13/5 (38.5)	4/2 (50.0)	4/3 (75.0)	4/3 (75.0) [ABM+] 4/4 (100.0) [XY+]

^**1**^PCR, Polymerase Chain Reaction

^2^qRT-PCR, quantitative Real Time-Polymerase Chain Reaction

^3^MDR, Multidrug-resistant profile

^**4**^XDR, Extensively drug-resistant profile

*Only 14 of the 47 isolates resistant to carbapenems were tested for qRT-PCR (10 MDR and 4 XDR).

Of 47 non-MBL carbapenem-resistant isolates, 14 were included in the qRT-PCR test for efflux pump overexpression, overproduction of AmpC and loss/reduction OprD porin (**Table 4**). The frequency of isolates with AmpC overproduction and loss of OprD porin was 71.4% (10/14) for both, and MexABOprM and MexXY pumps were 57.1% (8/14) and 64.3% (9/14), respectively. Most isolates with the MDR/XDR profile showed an association with three or more resistance mechanisms, and the presence of AmpC overproduction and loss of OprD porin predominated among non-MDR/XDR isolates that were only resistant to carbapenems (N = 4).

**Table 4:** Characterization of efflux pumps overexpression, overproduction of AmpC and loss/reduction OprD in *P*. *aeruginosa* isolates.

Isolates	Overproduction of AmpC		Overexpression of efflux pumps				Loss OprD	Resistance profile (MDR^1^/XDR^2^)
	D-Test^3^	AmpC	ABM	XY	EFN	CDJ	OprD	
**1**	-	+	-	+	-	-	+	CAZ^**4**^, PPTAZ^5^, IMI^6^, CIP^8^, LEV^9^, GEN^10^, AZT^11^ **(MDR)**
**2**	+	+	-	+	-	-	-	CPM^**12**^, IMI, MER^7^, CIP, GEN, AMK^13^, AZT **(MDR)**
**3**	+	-	+	+	-	-	+	CAZ, CPM, PPTAZ, IMI, MER, CIP, LEV, GEN, AMK, AZT **(XDR)**
**4**	+	-	+	-	-	-	+	CPM, IMI, MER, CIP, GEN, AMK, AZT **(MDR)**
**5**	+	+	+	+	-	-	+	CAZ, CPM, PPTAZ, IMI, MER, CIP, LEV, GEN, AZT **(XDR)**
**6**	+	+	+	+	-	-	+	CAZ, IMI, MER, CIP, LEV, GEN, AZT **(MDR)**
**7**	-	+	+	+	-	-	-	CAZ, CPM, IMI, MER, CIP, LEV, GEN, AMK **(MDR)**
**8**	+	+	+	-	-	-	+	IMP, MER (-)
**9**	+	+	-	-	-	-	+	IMP (-)
**10**	+	-	-	+	-	-	+	CAZ, CPM, IMI, MER, CIP, LEV, GEN, AMK, AZT **(XDR)**
**11**	-	-	+	+	-	-	-	CAZ, CPM, IMI, MER **(MDR)**
**12**	+	+	-	-	-	-	+	IMI (-)
**13**	+	+	-	-	-	-	+	IMI, MER (-)
**14**	+	+	+	+	-	-	-	CAZ, CPM, PPTAZ, IMI, MER, CIP, LEV, GEN **(XDR)**
**Total** **N (%)**	**11 (78,0)**	**10 (71,4)**	**8 (57,1)**	**9 (64,3)**	**0 (-)**	**0 (-)**	**10 (71,4)**	**-**

^**1**^MDR, Multidrug-resistant

^2^XDR, Extensively-resistant

^3^Phenotypic test for overproduction of AmpC

^**4**^Ceftazidime

^5^Piperacillin-tazobactam

^6^Imipenem

^7^Meropenem

^8^Ciprofloxacin

^9^Levofloxacin

^10^Gentamicin

^11^Aztreonam

^**12**^Cefepime

^13^Amicacin.

AmpC, Overproduction of AmpC; ABM, Overexpression of MexABOprM; XY, Overexpression of MexXY; EFN, Overexpression of MexEFOprN; CDJ, Overexpression of MexCDOprJ; OprD, Loss/alteration of OprD porin.

+ or–(positive or negative).

## Discussion

*Pseudomonas aeruginosa* is considered one of the most problematic pathogens among gram-negative bacteria that cause hospital infections, especially due to its extraordinary ability to acquire resistance genes [31,32]. In this study, we found that high rates of carbapenem-resistance, MDR and XDR profiles existed in *P*. *aeruginosa* clinical isoltes. A multicenter study in Latin America reported significant rates of reduced susceptibility to meropenem: 53.8% among the isolates from Argentina, 46.7% in Brazil, 33.3% in Chile and 28.8% of isolates from Mexico [8]. In Brazil, surveillance studies and independent research groups in the south and center of the country have reported an increasing trend in imipenem resistance rates for the years 2001, 2004 and 2009, at frequencies of approximately 30.2%, 58.9% and 82.7%, respectively [3,33,34]. In these cases, the use of antibiotic therapy was restricted to therapy with alternative drugs considered problematic due to their high toxicity, such as polymyxins (polymyxin B and colistin), that are often not commercially available in Brazil [35,36]. All isolates in our study were sensitive to polymyxin; however, the presence of clinical isolates with reduced susceptibility to this antimicrobial agent class has been reported in the literature [37,38].

Usually, the antibiotic use is high in developing countries, particularly in intensive care units [39,40]. This high consumption of antimicrobials results in a higher incidence of multidrug-resistant and extensively drug-resistant bacteria [41], combined with greater dissemination of these microorganisms [42]; this is explained, in part, by the lack of resources and failure to implement control practices and prevention [41]. The data from this study have verified most of the risk factors mentioned in the literature as those related to the acquisition of bacteremia by *P*. *aeruginosa* resistant to antimicrobials, including the over use of antimicrobials, lengthy hospital stays, admission to an intensive care unit, presence of invasive procedures, especially intravenous devices and mechanical ventilation, as well as a secondary source of infection, especially the lungs [43,44].

As previously mentioned, it is known that both the prior use of antimicrobials, as well as their use during hospitalization, are related to the increase in infections by resistant isolates [45,46]. Samonis *et al*. [47] demonstrated that the prior use of fluoroquinolones was independently associated with infection by extensively drug-resistant *P*. *aeruginosa* isolates. In this study, the use of broad-spectrum cephalosporins was predominant among the prescribed antibiotics, with large variations in the observed period. However, the use of carbapenems, including imipenem and meropenem, was also high. Additionally, in general, the antibiotics use in the Uberlandia University Hospital (UFU-HC) was much higher as compared to other countries [48], but without significant relationship with increased incidence of *P*. *aeruginosa* resistant to carbapenems in this study.

*P*. *aeruginosa* has a wide variety of intrinsic and acquired resistance mechanisms to different antimicrobials [2,13]. The carbapenem-resistance often results from the production of carbapenemases, particularly those that hydrolyze carbapenem, such as metallo-β-lactamases [2,4,5,49]. The prevalence of *P*. *aeruginosa* resistant to carbapenems phenotypically through production of MBL in Brazilian studies achieves rates above 50% in different geographic regions, and the SPM-1 enzyme is the most prevalent among the resistant isolates in the country [28,33]. However, other MBLs, including the VIM and IMP types, have also been identified in *P*. *aeruginosa* isolates in Brazil [26,37,49]. An important change in the epidemiology of *P*. *aeruginosa* has been observed in the UFU-HC, in which only producers SPM-1 were found from 2005 to 2011 [50] to the present, where our results indicate the emergence and spread of VIM.

When we evaluated the clonal relationship between strains containing the *bla*_SPM_ and *bla*_VIM_ genes, we observed high similarity (greater than 80%) among isolates containing *bla*_SPM;_ this was not observed for those containing the *bla*_VIM_ gene. The presence of a multidrug-resistant *P*. *aeruginosa* clone persisting for long periods in different hospital units reinforces the idea of resistance genes dissemination among hospitalized patients, emphasizing the need to improve prevention and control strategies of infection [51–53].

The carbapenem resistance mechanisms were investigated in greater detail in those isolates negative for MBL production, but no gene related to other carbapenemases was detected, and a positive result for MBL production was only observed through the Hodge test. These results suggest that in the absence of an efficient carbapenemase, other resistance mechanisms exist in these isolates, including overproduction of AmpC cephalosporinase, changes in permeability of the outer membrane through loss/reduction of the OprD porin and overexpression of efflux pumps.

Of all the efflux pumps already described in *P*. *aeruginosa*, MexABOprM, MexCDOprJ, and MexXY MexEFOprN are among the best-characterized systems, and they are associated with a variety of antibiotics resistance in clinical isolates [13,14]. In addition, they are known to be associated with multidrug-resistance, since one system can act on multiple substrates [54,55]. In the present study, all the isolates that tested positive in qRT-PCR and were characterized with overexpression of efflux pumps also exhibited some another type of associated resistance mechanism. In addition, it became apparent that the MDR phenotype, and/or especially the XDR phenotype, was present between the isolates that showed association of three or more resistance mechanisms, except the presence of MBL.

According to previous studies in Brazil, the efflux pumps most commonly found in clinical isolates of *P*. *aeruginosa* include MexABOprM and MexXY systems, which are constitutively expressed and play an important role in carbapenem-resistance and cover a wider range of antimicrobial resistance than the MexCDOprJ and MexEFOprN pumps [55,56]. In this study, no increased expression of *mexD* and *mexF* genes was observed among the isolates investigated, and the frequency of MexABOprM and MexXY overexpression was high. A recent study conducted on clinical isolates of multidrug-resistant *P*. *aeruginosa* from hospitals in Thailand revealed that 92.06% of those isolates overexpress the MexABOprM system and 63.49% overexpress the MexXY system [57]. In Brazil, another study evaluating this overexpression in clinical isolates of *P*. *aeruginosa* recovered from blood noted the presence of MexABOprM and MexXY expression in 27.1% and 50.8% of the isolates, respectively [26].

According to the literature, one of the most consistent findings for resistance to carbapenems, particularly imipenem, has been the impermeability of the membrane due to loss of the OprD porin [13,58–62], and several groups have reported rates of this type of mechanism exceeding 80% among clinical isolates of *P*. *aeruginosa* [26]. Here, we also noted significant rate reduction in the expression of the *oprD* gene. Moreover, the association between multidrug-resistance via loss of OprD in *P*. *aeruginosa* clinical isolates has been reported in literature, mainly as a synergistic effect with other resistance mechanisms [63]. In addiction, through this survey, this association has been well-characterized, as isolates with MDR/XDR and OprD porin loss also had concomitant overexpression of efflux pumps and hyperproduction of AmpC.

In conclusion, a high rate of resistance and multidrug-resistance among *Pseudomonas aeruginosa* isolates suggest some level of SPM-1 transmission that reinforces the importance of greater rigor in the prevention and control of infection in healthcare settings. Our results further confirm previous reports showing a high incidence of carbapenem-resistant *P*. *aeruginosa* in Brazilian hospitals and that this is associated with the abusive and indiscriminate use of antibiotics, indicating that it is also necessary to revise the antimicrobial use policies. Regarding the resistance mechanisms, our results showed that, in the absence of an effective carbapenemase, resistance to carbapenems in *P*. *aeruginosa* can be explained by overexpression of MexABOprM and MexXY systems, AmpC overproduction and loss of the OprD porin, that when presented in association with other factors can contribute to expression of MDR/XDR phenotypes.

## Supporting information

S1 Fig UPGMA dendrogram of PFGE profiles of 8 clinical *P*. *aeruginosa* isolates used in this study using the Dice coefficient under 1% tolerance and 1% optimization. A similarity coefficient of 80% was chosen for cluster definition. ^1^Metallo-β-lactamase.
(TIF)

S1 Table Relative gene expression of 14 *Pseudomonas aeruginosa* isolates in comparison to *P*. *aeruginosa* PAO1 reference strain. (DOCX)

S2 Table Defined daily dose of each antimicrobial/month, from June/2009 to December/2012. (DOCX)
