# Supplementary figures and images for: Molecular epidemiological survey of bacteremia by multidrug resistant Pseudomonas aeruginosa: the relevance of intrinsic resistance mechanisms
Source: PLoS One. 2017 May 8;12(5):e0176774. doi: 10.1371/journal.pone.0176774 (PMC5421754; doi:10.1371/journal.pone.0176774)

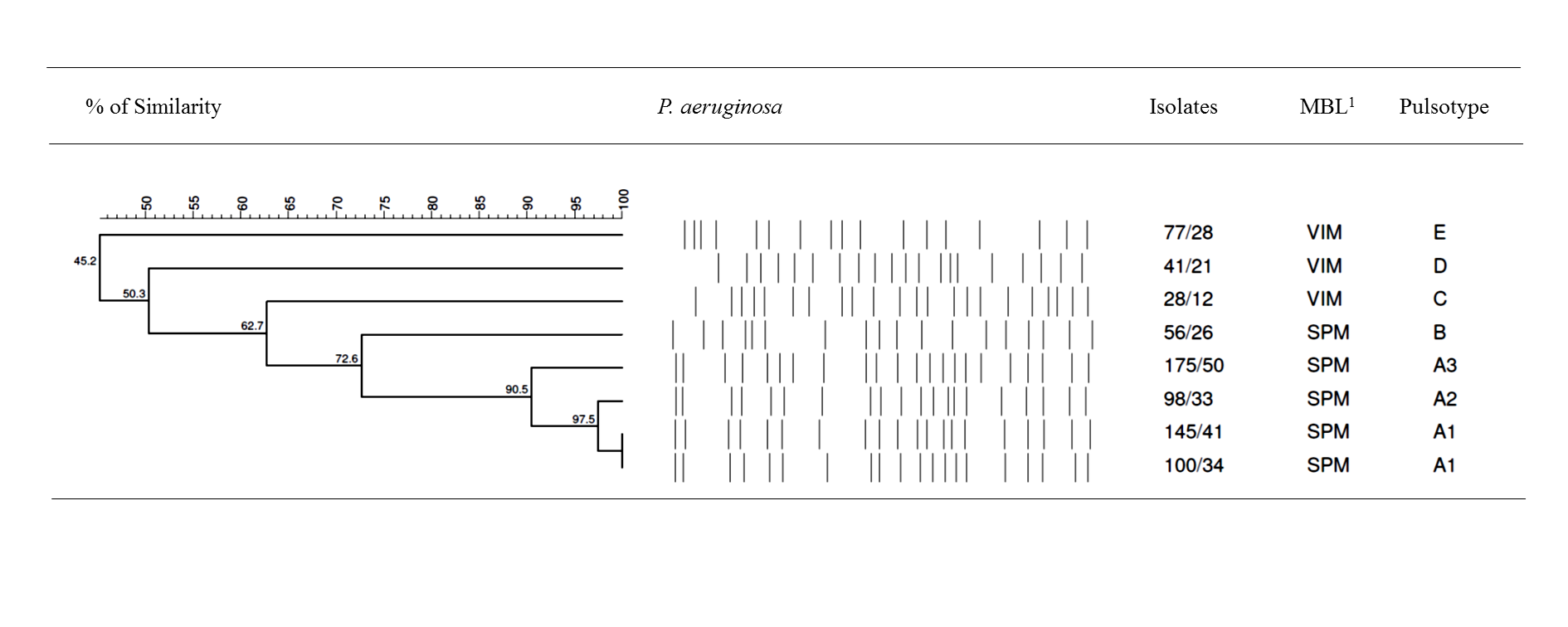

Supplement: S1 Fig — A similarity coefficient of 80% was chosen for cluster definition. 1Metallo-β-lactamase. (TIF) [file pone.0176774.s001.tif]
