# Supplementary material for: Molecular epidemiological survey of bacteremia by multidrug resistant Pseudomonas aeruginosa: the relevance of intrinsic resistance mechanisms
Source: PLoS One. 2017 May 8;12(5):e0176774. doi: 10.1371/journal.pone.0176774 (PMC5421754; doi:10.1371/journal.pone.0176774)
Supplement: S1 Table — (DOCX) [file pone.0176774.s002.docx]

**S1 Table.** **Relative gene expression of 14 *Pseudomonas aeruginosa* isolates in comparison to *P. aeruginosa* PAO1 reference strain**

| **Isolates** | **Genes** | | | | | |
| --- | --- | --- | --- | --- | --- | --- |
|  | ***mexB*** | ***mexY*** | ***mexF*** | ***mexD*** | ***ampC*** | ***oprD*** |
| **1** | 1,503 | 5,845 | 0,213 | 33,614 | 20,010 | 0,110 |
| **2** | 1,270 | 19,615 | 0,600 | 0,050 | 31,6011 | 1,650 |
| **3** | 6,448 | 14,029 | 5,516 | 1,124 | 0,476 | 0,040 |
| **4** | 28,112 | 0,073 | 18,614 | 0,006 | 9,793 | 0,003 |
| **5** | 52,167 | 34,088 | 85,678 | 1,475 | 90,141 | 0,149 |
| **6** | 5,399 | 13,453 | 9,202 | 2,875 | 65,343 | 0,241 |
| **7** | 24,063 | 53,449 | 3,118 | 22,816 | 258,339 | 0,879 |
| **8** | 30,036 | 1,810 | 0,182 | 0,254 | 140,672 | 0,182 |
| **9** | 2,061 | 1,342 | 0,938 | 5,752 | 273,420 | 0,015 |
| **10** | 1,194 | 12,337 | 25,315 | 11,246 | 9,104 | 0,035 |
| **11** | 58,556 | 10,945 | 3,913 | 0,295 | 5,782 | 1,253 |
| **12** | 1,744 | 3,754 | 2,919 | 0,114 | 236,811 | 0,043 |
| **13** | 1,690 | 1,086 | 12,556 | 3,215 | 191,028 | 0,001 |
| **14** | 64,729 | 40,850 | 34,478 | 29,681 | 239,933 | 0,737 |
