# Supplementary material for: Molecular epidemiological survey of bacteremia by multidrug resistant Pseudomonas aeruginosa: the relevance of intrinsic resistance mechanisms
Source: PLoS One. 2017 May 8;12(5):e0176774. doi: 10.1371/journal.pone.0176774 (PMC5421754; doi:10.1371/journal.pone.0176774)
Supplement: S2 Table — (DOCX) [file pone.0176774.s003.docx]

**S2 Table. Defined Daily Dose of each antimicrobial/month, from June/2009 to December/2012**

| **Period** | **Imipenem** | **Meropenem** | **Cefepime** | **Ceftriaxone** | **Ciprofloxacin** | **Levofloxacin** |
| --- | --- | --- | --- | --- | --- | --- |
| June 2009 | 16.3 | 27.1 | 95 | 74.8 | 6.0 | 3.2 |
| July 2009 | 8.9 | 62.2 | 110.3 | 84.8 | 6.1 | 2.3 |
| August 2009 | 10.4 | 82.7 | 138.6 | 88.3 | 5.0 | 1.6 |
| September 2009 | 17.4 | 59.1 | 105.1 | 80 | 4.0 | 1.7 |
| October 2009 | 15.2 | 32.9 | 119.4 | 95.4 | 5.6 | 1.2 |
| November 2009 | 15 | 36.5 | 88.3 | 87.5 | 9.9 | 1.1 |
| December 2009 | 17.9 | 23.3 | 127.9 | 65.9 | 9.5 | 0.7 |
| January 2010 | 24.4 | 22.8 | 138.2 | 82.3 | 9.4 | 7.0 |
| February 2010 | 23.1 | 26.4 | 131.1 | 73.4 | 5.3 | 2.9 |
| March 2010 | 18.4 | 28.5 | 132.8 | 89.7 | 14.3 | 3.6 |
| April 2010 | 26.9 | 13.8 | 133.6 | 86.2 | 7.1 | 9.2 |
| May 2010 | 22.6 | 20.7 | 174.3 | 94.4 | 12.2 | 3.1 |
| June 2010 | 18.3 | 24.1 | 150.2 | 80.4 | 10.6 | 3.7 |
| July 2010 | 23.3 | 27.5 | 143.6 | 91.4 | 12.5 | 3.3 |
| August 2010 | 23.4 | 38.3 | 122 | 89.6 | 9.5 | 3.2 |
| September 2010 | 22.6 | 19.3 | 179.2 | 100.5 | 10.8 | 5.1 |
| October 2010 | 25.9 | 28.6 | 182.8 | 101.4 | 13.9 | 5.3 |
| November 2010 | 28.6 | 29.5 | 138.1 | 92.5 | 5.3 | 4.1 |
| December 2010 | 16.8 | 26.7 | 120.4 | 87.1 | 13.1 | 3.6 |
| January 2011 | 21.4 | 24.1 | 136.4 | 84.8 | 10.71 | 5.1 |
| February 2011 | 18.9 | 23.5 | 137.4 | 76.3 | 11.16 | 4.4 |
| March 2011 | 15.5 | 27.5 | 149.8 | 71.6 | 13.11 | 2.2 |
| April 2011 | 29.3 | 19.6 | 175.7 | 86.5 | 9.13 | 1.8 |
| May 2011 | 16.5 | 25.3 | 181.4 | 90.3 | 8.86 | 1.3 |
| June 2011 | 19.5 | 28.8 | 157.8 | 78.1 | 5.69 | 1.2 |
| July 2011 | 20.6 | 27.4 | 120.5 | 75.9 | 7.3 | 0.63 |
| August 2011 | 18.7 | 36.8 | 134.4 | 83 | 9.86 | 0.79 |
| September 2011 | 14.6 | 19.9 | 140.6 | 80.7 | 12.5 | 1.5 |
| October 2011 | 17.1 | 28.8 | 159.6 | 80.9 | 10.09 | 0.94 |
| November 2011 | 13 | 19.3 | 125.3 | 76.9 | 11.18 | 2.1 |
| December 2011 | 17.1 | 19.5 | 152.8 | 84.8 | 14.5 | 0.69 |
| January 2012 | 26.9 | 32.9 | 224.8 | 99.7 | 22.7 | 11.0 |
| February 2012 | 29.5 | 27.6 | 170.8 | 72.8 | 15.1 | 6.0 |
| March 2012 | 23.3 | 31.2 | 203.1 | 101.7 | 28.7 | 10.9 |
| April 2012 | 14.2 | 14.9 | 153.4 | 84.9 | 9.3 | 5.8 |
| May 2012 | 19.2 | 27.3 | 254.1 | 118.5 | 12.2 | 11.8 |
| June 2012 | 12.0 | 20.7 | 63.6 | 118.8 | 17.9 | 10.4 |
| July 2012 | 22.7 | 33.4 | 103.1 | 101.1 | 17.4 | 15.4 |
| August 2012 | 17.4 | 50.9 | 155.1 | 107.2 | 19.1 | 11.6 |
| September 2012 | 17.9 | 37.7 | 226.7 | 120.9 | 15.5 | 9.0 |
| October 2012 | 16.8 | 45.6 | 159.7 | 109.5 | 20.3 | 8.5 |
| November 2012 | 18.7 | 49.9 | 212.9 | 113.7 | 26.8 | 13.7 |
| December 2012 | 13.9 | 34.1 | 147.7 | 83.1 | 11.2 | 6.9 |
